# Suitcase Lab: new, portable, and deployable equipment for rapid detection of specific harmful algae in Chilean coastal waters

**DOI:** 10.1007/s11356-020-11567-5

**Published:** 2020-11-18

**Authors:** So Fujiyoshi, Kyoko Yarimizu, Yohei Miyashita, Joaquín Rilling, Jacquelinne J. Acuña, Shoko Ueki, Gonzalo Gajardo, Oscar Espinoza-González, Leonardo Guzmán, Milko A. Jorquera, Satoshi Nagai, Fumito Maruyama

**Affiliations:** 1 Office of Industry-Academia-Government and Community Collaboration, Hiroshima University, 1-3-2 Kagamiyama, Higashi-Hiroshima City, Hiroshima 739-8511 Japan; 2 Center for holobiome and built Environment (CHOBE), Hiroshima University, 1-3-2 Kagamiyama, Higashi-Hiroshima City, Hiroshima 739-8511 Japan; 3 Laboratorio de Ecología Microbiana Aplicada, Departamento de Ciencias Químicas y Recursos Naturales, Scientific and Biotechnological Bioresource Nucleus (BIOREN-UFRO), Universidad de La Frontera, Ave. Francisco Salazar 01145, Temuco, Chile; 4 Institute of Plant Science and Resources, Okayama University, 2-20-1 Chuo, Kurashiki, Okayama 710-0046 Japan; 5 Laboratorio de Genética, Acuicultura & Biodiversidad. Departamento de Ciencias Biológicas y Biodiversidad, Universidad de Los Lagos, Osorno, Chile; 6 Centro de Estudios de Algas Nocivas (CREAN), Instituto de Fomento Pesquero (IFOP), Padre Harter 547, 5480000 Puerto Montt, Chile; 7 Instituto de Fomento Pesquero, IFOP, Balmaceda 252, 5480000 Puerto Montt, Chile; 8 Japan Fisheries Research and Education Agency, Fisheries Stock Assessment Center, Bioinformatics and Biosciences Division, Genome Structure Analysis Group, 2-12-4 Fukuura, Kanazawa-ku, Yokohama, Kanagawa 236-8648 Japan

**Keywords:** Deployable toolkit, Loop-mediated isothermal amplification, On-site molecular detection, Harmful algal bloom, Plankton monitoring

## Abstract

Phytoplankton blooms, including harmful algal blooms (HABs), have serious impacts on ecosystems, public health, and productivity activities. Rapid detection and monitoring of marine microalgae are important in predicting and managing HABs. We developed a toolkit, the Suitcase Lab, to detect harmful algae species in the field. We demonstrated the Suitcase Lab’s capabilities for sampling, filtration, DNA extraction, and loop-mediated isothermal amplification (LAMP) detection in cultured *Alexandrium catenella* cells as well as Chilean coastal waters from four sites: Repollal, Isla García, Puerto Montt, and Metri. A LAMP assay using the Suitcase Lab in the field confirmed microscopic observations of *A. catenella* in samples from Repollal and Isla García. The Suitcase Lab allowed the rapid detection of *A. catenella*, within 2 h from the time of sampling, even at a single cell per milliliter concentrations, demonstrating its usefulness for quick and qualitative on-site diagnosis of target toxic algae species. This method is applicable not only to detecting harmful algae but also to other field studies that seek a rapid molecular diagnostic test.

**Supplementary Information:**

The online version contains supplementary material available at 10.1007/s11356-020-11567-5.

## Introduction

Phytoplankton blooms are frequent phenomena observed in coastal regions of every continent in the world. Some of the blooming species have been found to produce endogenous toxins and such harmful algae blooms (HABs) can, directly and indirectly, cause acute effects on marine and freshwater ecosystems leading to major impacts on public health and productivity activities such as aquaculture, fisheries, and tourism (Lewitus et al. 2012). The frequency of HABs in Chile has also been increasing over the decades following a global trend. In particular, a bloom of *Pseudochattonella verruculosa* occurred during February and March of 2016 in Chile causing record-high economic damages to the country. That HAB event killed 39 million salmon mostly in Reloncavi Sound and Fjord (Anderson et al. 2016; Díaz et al. 2019; León-Muñoz et al. 2018). Subsequently, a prolonged bloom of *Alexandrium catenella* occurred in the same year in Chile, which caused paralytic shellfish poisoning in higher vertebrates from the inner sea of Chiloé Island to the far north (Eckford-Soper and Daugbjerg 2016a; Montes et al. 2018; Paredes et al. 2019). The Chilean government, in conjunction with research institutes, has promoted an early warning HAB species detection system to mitigate the effects of these HABs as they emerge. One of the issues is that the complete mechanism of a HAB is not fully understood, and thus a successful model has not been established to aid in avoiding HAB damages.

The current strategy to reduce the HAB related damages relies on early detection of HAB species and toxin levels by frequent coastal monitoring. Microscopy to identify and quantify HAB species is traditionally the standard monitoring tool to assess and manage regional ecosystems. However, microscopy miss detection of HAB species when they are at low abundance during pre-bloom periods or their morphology is transformed by the use of cell-fixing agents such as Lugol’s solution and paraformaldehyde (John et al. 2005; Zingone et al. 2006; Rodríguez-Ramos et al. 2013). Additionally, microscopy requires specialized and trained taxonomists that routinely analyze high numbers of samples (Orozco and Medlin 2013). More recently, satellite imaging and molecular methods have come into play to monitor HABs in conjunction with microscopy. Satellite observation provides the great advantage of obtaining real-time information on chlorophyll concentrations in any aquatic environment in the world (Hu 2009). The drawback of this method is that it cannot identify a bloom species nor can it determine which family of organisms is responsible for the chlorophyll value in the target aquatic regions. Molecular methods, such as fluorescent hybridization assays, sandwich hybridization assays, automatized biosensor detections, and real-time PCR assays, are superior in their ability to identify and quantify HAB species even at low abundance (Herrera-Sepulveda et al. 2013; Penna and Galluzzi 2013). However, they have some disadvantages, including difficulty in transportation, time-consuming sample processing, expensive equipment and reagents, cold-chain requirements, and the need to be conducted in specialized laboratories. A successful HAB monitoring tool can be best established from not a single tool but a combination of multiple tools considering each tool’s pros and cons. We focus on a rapid, accurate, and cost-effective approach to detect toxic algae in situ, directly in aquatic environments, to provide quick screening information on HABs. Here, we introduce our development of a portable molecular diagnostic laboratory system, called the “Suitcase Lab,” for quick screening of toxic alge.

Use of the portable laboratory systems such as PHAC-NML mobile laboratory (National Microbiology Laboratory of the Public Health Agency of Canada), Lab Without Walls (Lab Without Walls Inc., Australia), and MinION (Oxford Nanopore Technologies, UK) have been highly recognized in the past for rapid on-site detection of life-threatening microorganisms such as Zika virus, Ebola virus, N1H1 influenza, and *Burkholderia pseudomallei*, the bacteria that cause melioidosis (Inglis et al. 2011; Grolla et al. 2012; Inglis 2013; Faria et al. 2017; Quick et al. 2016). These outbreaks in many cases occur in regions with a limited source of health specialists, infrastructure, and logistics, such as poorly serviced roads and air systems. Therefore, introducing a portable laboratory in such isolated areas is a valuable strategy to rapidly diagnose critical cases to support patient management and surveillance. With the same concept, the Suitcase Lab was developed for HAB detection to be used in geographically complex areas such as the southern region of Chile where most HAB damages are reported (Montes et al. 2018). The common monitoring procedures in Chile include multi-day sailing to collect samples from several stations and sent to the closest testing laboratory by bus for microscopy. Thus, it can take days before the HAB species are identified and reported to appropriate health authorities. Fast detection time is critical for many HAB species, as exemplified in *Pseudo-nitzschia australis* that produce domoic acid, a highly potent toxin, to rapidly reach the regulatory limit (Díaz et al. 2019). Currently, no adequate in situ screening system exists for quick qualitative diagnoses of HABs. To remedy this, we developed the Suitcase Lab which, as its name suggests, is small enough to be contained within a single-wheeled suitcase. It includes a portable loop-mediated isothermal amplification (LAMP) device that can both provide a thermoblock system as well as a quantitative fluorometer. Supporting materials include sterile tubes, pipettes, and filtration units needed for environmental water sampling (cite table of lab contents).

Genetic testing technology based on LAMP has a wide range of applications. It enables the detection of target DNA or RNA by a reverse transcription-LAMP reaction rapidly, accurately, and inexpensively (Tomita et al. 2008; Mori et al. 2013). The method uses a specially designed set of target-specific primers: forward inner primer (FIP), back inner primer (BIP), two primers (F3 and B3), loop primer forward (LF), and loop primer reverse (LB). The first four primers recognize six regions on the target DNA, increasing target specificity to preclude false positives (Tomita et al. 2008), and the latter two loop primers accelerate the LAMP reaction (Nagamine et al. 2002). This LAMP amplification reaction uses only a small amount of DNA and proceeds at a constant temperature (60–65 °C) without requiring an additional expensive device, such as a thermal cycler (Notomi et al. 2000).

In the present study, we introduce the Suitcase Lab by (1) validating the results of the LAMP assay on cultured *A. catenella*, a well-known HAB species, (2) demonstrating the adaptability of the portable laboratory to detect *A. catenella* in field samples collected from Chilean coastal waters, and (3) comparing species detection data obtained by the Suitcase Lab with those from traditional microscopy.

## Materials and methods

### Suitcase Lab

**Fig. 1:**
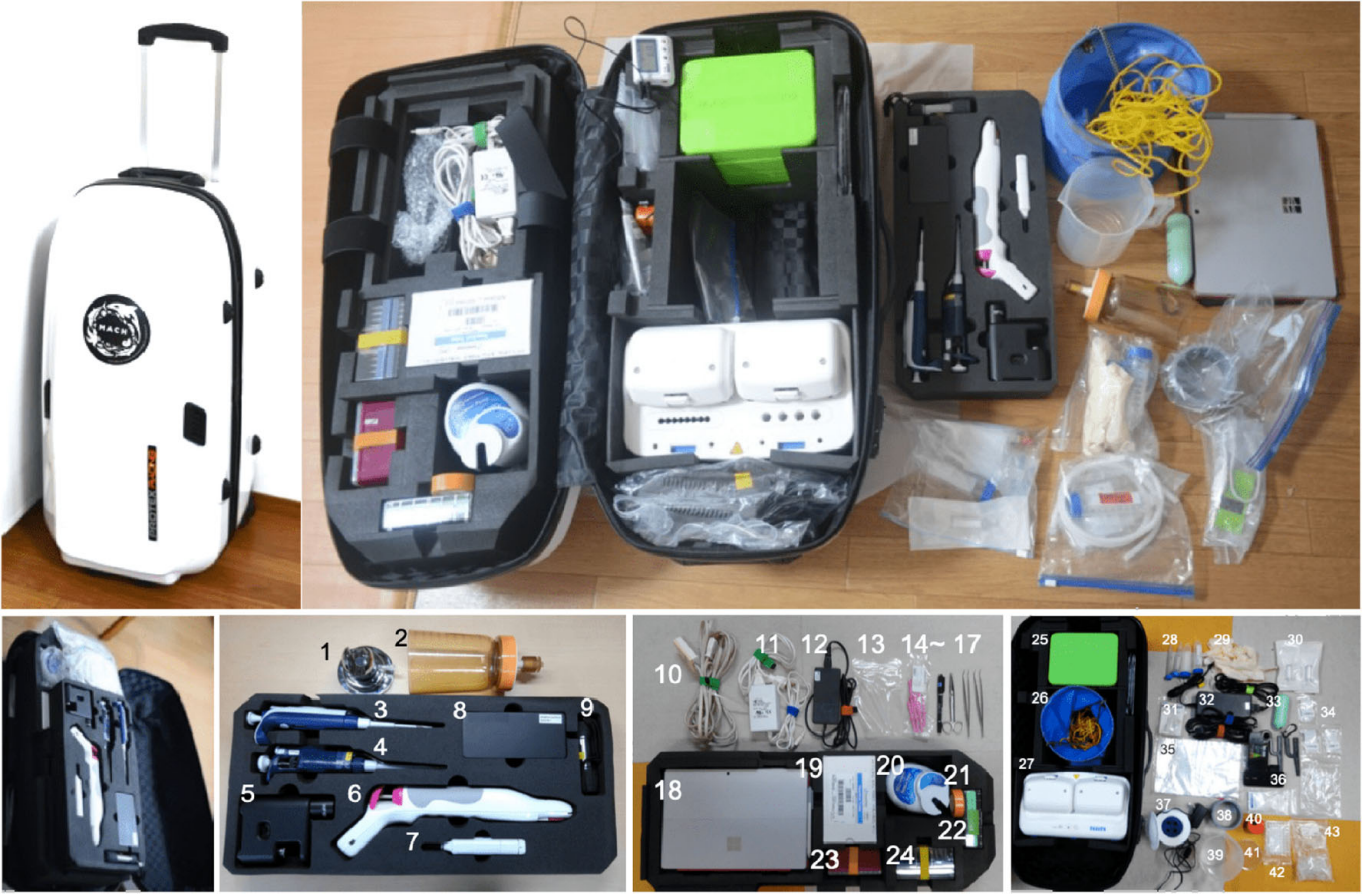
The Suitcase Lab. The Suitcase Lab is 47 × 39 × 90 cm (W × D × H) and weights 15 kg. It contains 43 tools

The Suitcase Lab developed in this study contains LAMP device and basic laboratory materials such as micropipettes, pipette tips, tubes, microscope (MJ-396, Sato Shoji Corporation, Tokyo, Japan), cool box (CoolBox™ XT PCR Strip Workstation, BioCision, CA, USA) with a cooling agent (XT Cooling core, BioCision), pump (Sentino® Microbiology pump, PALL, MI, USA), and thermostatic color sensor (MyAbscope, KANEKA, Osaka, Japan) (Fig. 1 and Table 1). The lab materials, including the filter unit, were autoclaved prior to packing in the Suitcase Lab as needed. The primers used for the LAMP assay (Table 2) were stored with a pre-chilled cooling agent at 4 °C in the cold box (No. 25 in Fig. 1) enclosed in the Suitcase Lab. A prior study reported that particularly environmental DNA tends to degrade at a temperature higher than 5 °C after a day and strongly suggested a proper storage condition for samples as well as reagent used for field studies (Eichmiller et al. 2016). Therefore, to evaluate how long the cooling agent could last in the cold box, the temperatures inside and outside the cold box were recorded with a temperature data logger (TR-71wf, T&D, Nagano, Japan) in a standard lab to verify temperature changes during the period of the experiment for 24 h.

**Table 1:** Contents of the Suitcase Lab

No.	Name	Company (city, country)	Cat. no.	Voltage
1	Funnel holder	–	–	NA
2	Magnetic filter funnels, 47 mm, 500 mL capacity	PALL (MI, USA)	4277	NA
3	Micropipette (2–20 μL)	–	–	NA
4	Micropipette (20–200 μL)	–	–	NA
5	Microscope (MJ-396)	Sato shoji Corporation (Tokyo, Japan)	MMS-020-01	Battery
6	Handheld automated cell counter	Merck Millipore (Darmstadt, Germany)	PHCC20060	Battery
7	Masher [The masher in Fig. 1 (No.7) is no longer available]	Nippi Inc. (Tokyo, Japan)	Instead: Power masherII (891,300, Nippi Inc.)	Battery
8	Electrical outlet for PC	–	–	NA
9	UV LED light	–	–	Battery
10	Extension cord	–	–	NA
11	Electrical outlet for pump	PALL (MI, USA)	13,186	NA
12	Electrical outlet for PC	–	–	NA
13	Disposable pipette	–	–	NA
14	Homogenizer pestle	–	–	NA
15	Marker	–	–	NA
16	Scissors	–	–	NA
17	Tweezers	–	–	NA
18	PC with Windows OS [In this study, Microsoft Surface Pro 4 TH2-00014 was used.]	–		–
19	Tube (200 μL)	–	–	NA
20	Suction pump	PALL (MI, USA)	13,186	Battery or 100–240 VAC
21	Adhesive tape	–	–	NA
22	200-μL tube stand	–	–	NA
23	Pipette tips (20 μL)	–	–	NA
24	Pipette tips (200 μL)	–	–	NA
25	Cold box with cooling agent	BioCision. (CA, USA)	BCS-572	NA
26	Bucket with rope	–	–	NA
27	Thermostatic color sensor	KANEKA (Osaka, Japan)	KN-T100901	19 VDC
28	Falcon tube (50 ml, 15 ml)	–	–	NA
29	Disposable gloves	–	–	NA
30	Filter unit (0.2 μm)	Merck Millipore (Darmstadt, Germany)	SVGP01050	NA
31	DNA extraction reagents	KANEKA (Osaka, Japan)	KN-T110005	NA
32	Electrical outlet for thermostatic color sensor	KANEKA (Osaka, Japan)	KN-T100901	NA
33	Weight (5 kg)	–	–	NA
34	Parts for pump	PALL (MI, USA)	13,186	NA
35	Plastic bags	–	–	NA
36	Microscope’s accessories	Sato shoji Corporation (Tokyo, Japan)	MMS-020-01	NA
37	Centrifuge	Waken Btech co. Ltd. (Kyoto, Japan)	WKN-2374	100–240 VAC
38	10 μm Nylon mesh sieve	–	–	NA
39	Plastic beaker with handle	–	–	NA
40	Cell counter	–	–	NA
41	Petri dish	–	–	NA
42	PCR tube (200 μL)	–	–	NA
43	1.5 mL tube	–	–	NA

*–*, unspecified; *NA*, not applicable

**Table 2:** Primer sequence

Species	Target region	Primer name	Primer sequence	Length (bp)	Ref.
*Alexandrium pacificum* (accession no. AB565484, previously named *A. catenella*)	28S-rDNA (D1/D2)	Acat406-F3-2	GAGATTGTAGTGCTTGCTTA	20	(Nagai and Itakura 2012)
		Acat406-B3	AAGCAACCTCAAGGACAAG	19	
		Acat406-FIP	TGTCCACATAAAAACTGGCACAACAATGGGTTTTGGCTGCAA	42	
		Acat406-BIP	GGTAATTTTCCTGCGGGGTGTGGGACACAAACAAATACACCAG	43	
		Acat406-LF2	GCAAGAATTATTGCAC	16	
		Acat406-LB2	GCATGTAATGATTTGCATGTT	21	
*Alexandrium catenella* (accession no. AB565483, previously named *A. tamarense*)	28S-rDNA (D1/D2)	AT282-F3	TGGTGGGAGTGTTGC	15	
		AT282-B3	AAGTCCAAGGAAGGAAGC	18	
		AT282-FIP	AGAAACTGGCATGCAAAGAAAGACTTGCTTGACAAGAGCT	40	
		AT282-BIP	GTCTCCTGTGGGGGGTGGATTGATCCCCAAGCACAGGAAC	40	
		AT282-LF	AATCATTACACCCACAGCCC	20	
		AT282-LB	AATGTGTCTGGTGTATGTGTG	21	

### Sample information and DNA extraction

#### Culture samples

All procedures were performed in a standard lab. Clonal strains of *A. pacificum* (isolated from Ago Bay, Mie Pref. Japan, accession no. AB565484, previously named *A. catenella*) and *A. catenella* (isolated from Hiroshima Bay, Hiroshima Pref. Japan, accession no. AB565483, previously named *A. tamarense*) were kindly shared by Dr. Hiroshi Oikawa (Japan Fisheries Research and Education Agency) and one of the authors, SN, respectively. They were individually grown in 25 mL of Daigo IMK medium (Wako, Tokyo, Japan). Using the Easy DNA Extraction Kit version2 (Kaneka, KN-T110005) for its quick and simple procedure, DNA was extracted from the culture cells according to the manufacture’s protocol: Briefly, 100 μL of alkaline “solution A” was added to a sample, incubated at 98 °C for 8 min, and then neutralized with 14 μL of “solution B” to make the final solution. The extracted DNA was used for LAMP assays.

#### Field tests

A mock study was performed in Takehara Port (Hiroshima prefecture, Japan) to test the usefulness of the Suitcase Lab. All the Suitcase Lab equipment was packed in a standard lab, and then all procedures stated here after were conducted in the field. Gloves were worn at all times to maintain experimental conditions as clean as possible. Five hundred millimeters of water was collected in a portable bucket pre-cleaned with in situ seawater 2~3 times. The water was filtered through a 10-μm nylon mesh sieve (No. 38 in Fig. 1), and the residue on the sieve (about 1–1.5 mL) was transferred to a 1.5-mL sterile tube with a sterile disposable pipette. The tube containing seawater was then centrifuged at 420–950×*g* (No. 37 in Fig. 1, Petit Spin, WakenBtech, Kyoto, Japan) for 15 min and the supernatant was discarded. The DNA was extracted from the pellet using the Easy DNA Extraction Kit version 2, according to the manufacturer’s protocol. LAMP assays were performed using 1 μL of each of six primers for *A. catenella* (Table 2), 10 μL of template DNA, 9 μL of nuclease-free water. The LAMP sample mixture was added to a Dried RNA/DNA Amplification Reagent tube (Eiken Chemical Co., Ltd., Tokyo, Japan). The same amount of nuclease-free water as the DNA samples (10 μL) was used as a negative control. The LAMP assay was conducted using the thermostatic color sensor (No. 27 in Fig. 1, MyAbscope, KANEKA, Osaka, Japan) with incubation of 62 °C for 30 min, deactivation of 80 °C for 2 min, and turbidity measurement at wavelength of 575–660 nm (R color setting in the operating system) in real time.

### Specificity of the LAMP assay using algal cells in laboratory culture

The specificity of the LAMP assay was tested from the algal cultures in a standard lab using *A. catenella* as the target species. As described in “Sample information and DNA extraction,” the DNA was extracted from a laboratory culture of *A. catenella* (ca. 4075 cells mL^−1^) and *A. pacificum* (ca. 650 cells mL^−1^). Six primers for *A. catenella* designed and validated by Nagai and Itakura (2012) were used here (Table 2). The LAMP sample mixture was prepared according to Table 3, using the LoopAmp DNA amplification kit (Eiken Chemical Co., Ltd., Tokyo, Japan). Specifically, each reaction contained 12.5 μL of × 2 reaction mix, 1 μL of each of six primers, 1 μL of *Bst* DNA polymerase (8 U), 2 μL of template DNA, 1 μL of fluorescent detection reagent (Loopamp Fluorescent Detection Reagent; Eiken Chemical Co., Ltd., Tokyo, Japan), and adding nuclease-free water to a final volume of 25 μL (Nagai et al. 2012). The LAMP assay was conducted using the thermostatic color sensor (No. 27 in Fig. 1, MyAbscope, KANEKA, Osaka, Japan) with incubation of 62 °C for 30 min, deactivation of 80 °C for 2 min, and turbidity measurement at wavelength of 575–660 nm (R color setting in the operating system) in real time.

**Table 3:** Materials and solution for Loop-mediated isothermal amplification (LAMP) reaction

Reagent	Liquid type		Reagent	Lyophilized type	
	μL/reaction	Cost per reaction (US$)		μL/reaction	Cost per reaction (US$)
× 2 reaction mix	12.5	–	Lyopilized reagent	–	5.65
BST DNA polymerase	1	3.72^*1^	Distilled water	9	–
Distilled water	2.5	–	FIP primer (40 μM)	1	0.04
FIP primer (40 μM)	1	0.04	BIP primer (40 μM)	1	0.04
BIP primer (40 μM)	1	0.04	F3 primer (5 μM)	1	0.03^*2^
F3 primer (5 μM)	1	0.03^*2^	B3 primer (5 μM)	1	0.03^*2^
B3 primer (5 μM)	1	0.03^*2^	Loop primer-F (20 μM)	1	0.02
Loop primer-F (20 μM)	1	0.02	Loop primer-R (20 μM)	1	0.02
Loop primer-R (20 μM)	1	0.02	Template DNA	10	Depending on your sample
Fluorescent reagent	1	0.73			
Template DNA	2	Depending on your sample			
Total	25	4.63		25	5.83

^*1^×2 reaction mix, BST DNA polymerase and distilled water were contained in Loop Amp DNA Amplification (Eiken Chemical Co., Ltd., Tokyo, Japan)

^*2^The price of 42-base oligo amplification was US$9. This can be used for about 250 reactions, resulting in a price of US$0.036 reaction^−1^

### Sensitivity of the LAMP assay using algal cells in laboratory cultures

The sensitivity of the LAMP assay was tested from the algal cultures in a standard lab. *A. catenella* culture containing ca. 4075 cells mL^−1^ was used as a positive control and nuclease-free water was used as a negative control. DNA was extracted from 500 μL of each *A. catenella* dilution (10^−1^, 10^−2^, 10^−3^, 4 × 10^−3^, and 10^−4^), which theoretically contained cell counts of ca. 407.5, 40.8, 4.08, 1.02, and 0.41 cells mL^−1^, respectively. The LAMP assay was performed for each sample DNA using the method described in “Specificity of the LAMP assay using algal cells in laboratory culture.”

### Specific detection of Chilean coastal water samples by microscopy and LAMP

**Fig. 2:**
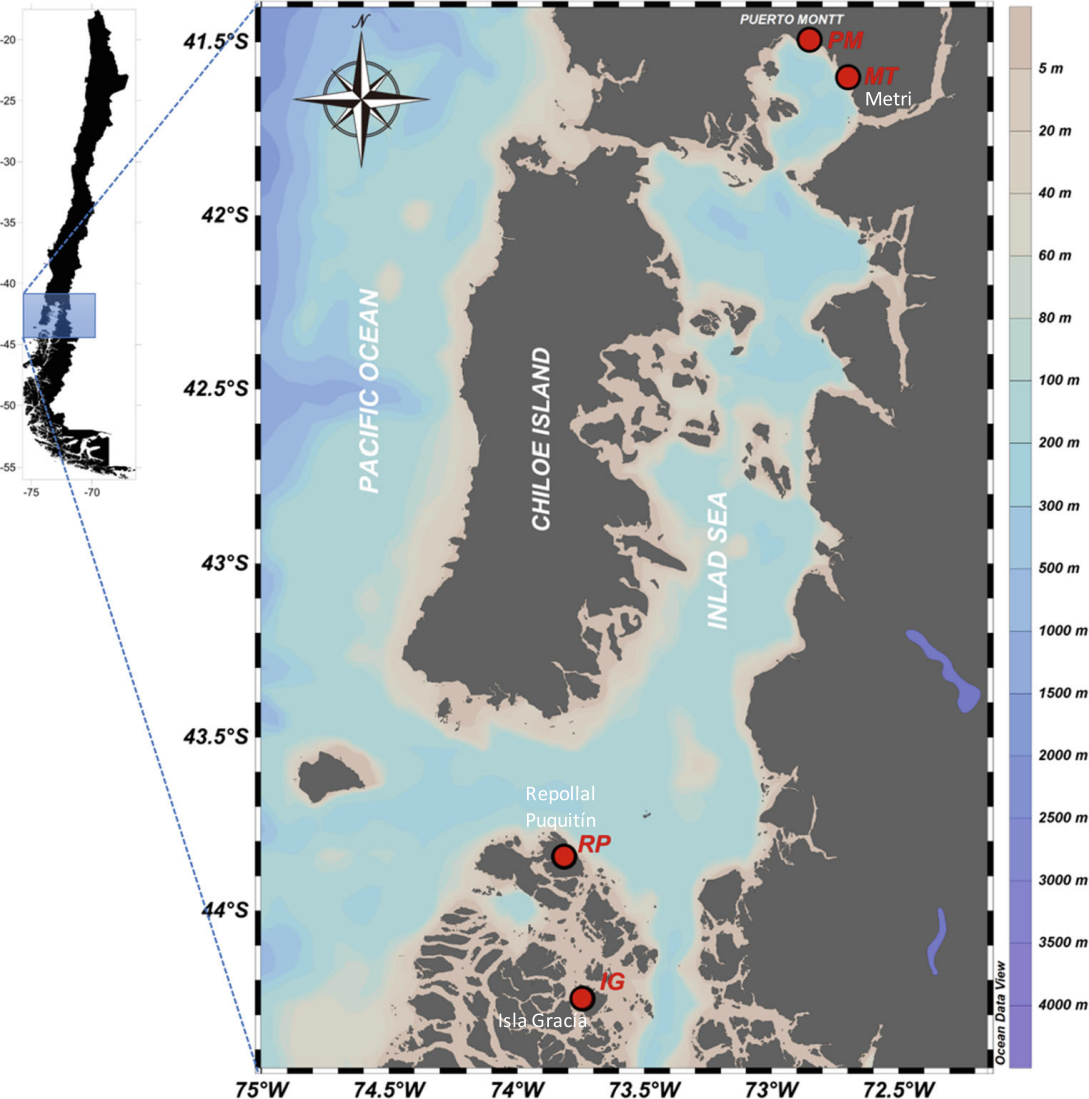
Sampling locations. Chilean coastal water samples were collected from four locations: Metri (MT) (41° 60′ S), Puerto Montt (PM) (41° 27′ S), Repollal Puquitín (RP) (43° 45′ S), and Isla García (IG) (44° 15′ S). The sampling locations were plotted in Ocean Data View (Schlitzer 2002)

For microscopy, six samples (200 L sample^–1^) were taken from depths of 0–20 m with a 23-μm phytoplankton net and pooled so that the final volume filtered by the net in these six samples was around 1200 L. Using the Suitcase Lab, a pilot sampling was performed on the Chilean coast, and the assay and analysis of the field samples were done using the enclosed LAMP device: Coastal water samples were collected from four locations: Metri (MT) (41° 60′ S); Puerto Montt (PM) (41° 27′ S), Repollal Puquitín (RP) (43° 45′ S), and Isla García (IG) (44° 15′ S) in February 2019 (Fig. 2). Two hundred fifty milliliters of each seawater was filtered through a 0.22-μm filter (Sterivex, Merck Millipore, Darmstadt, Germany). The filtered membranes were cut in half, one to freeze as a back-up sample and the other to slice into pieces in a 2.0-mL tube using sterilized medical scissors to proceed DNA extraction. To the tube containing the sliced membrane, 500 μL of 5% Chelex (Chelex 100 Chelating resin, Bio-Rad, Hercules, CA) was added, and the membranes were homogenized with a masher (Power masher Nippi Inc. Tokyo, Japan) and a homogenizer pestle (Violamo homogenizer pestle R1.5, Azone, Osaka, Japan) for 5 min to break the cells. Then, the tube was heated for 20 min at 97 °C for DNA extraction (Nagai et al. 2012). The product was centrifuged (Petit Spin, WakenBtech, Kyoto, Japan), and the supernatant was transferred to a new tube. LAMP assays were performed as described above, using 1 μL of each of six primers for *A. catenella*, 10 μL of template DNA, nuclease-free water to a final volume of 25 μL, and added to a Dried RNA/DNA Amplification Reagent tube (Eiken Chemical Co., Ltd.). The same amount of nuclease-free water as the DNA samples (10 μL) was used as a negative control.

## Results and discussion

### Suitcase Lab

**Fig. 3:**
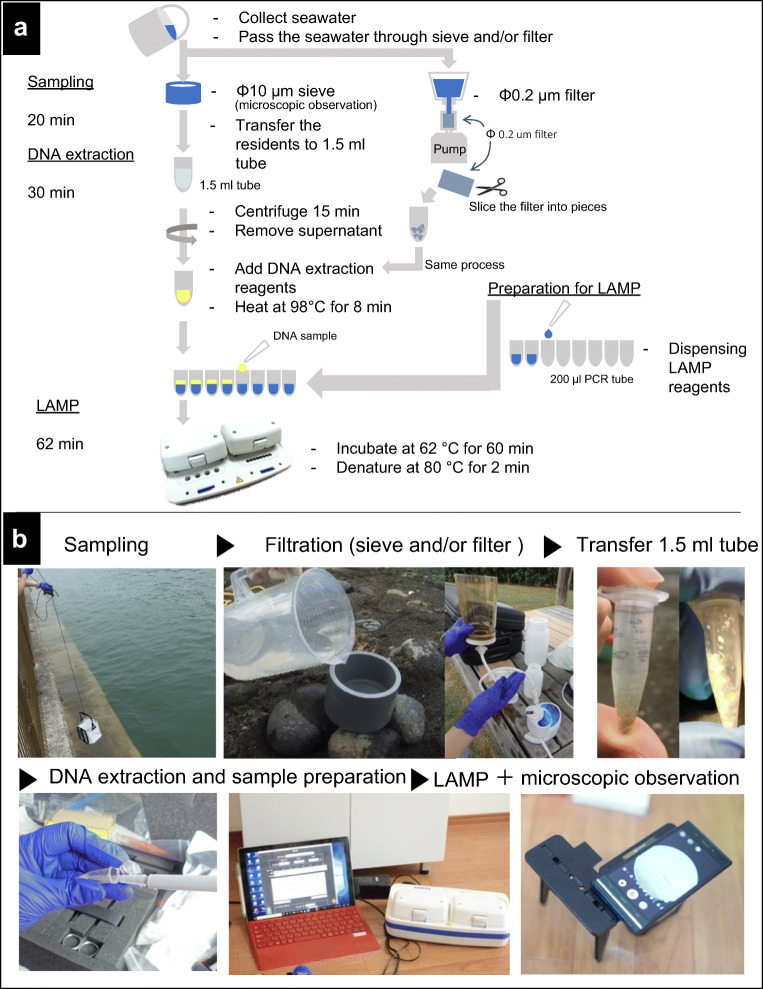
Schematic DNA extraction procedure for Loop-mediated isothermal amplification (LAMP) assay (**a**), and sample collection, treatment and analysis on site (**b**)

We developed the Suitcase Lab by focusing on easy on-site handling and operation for HAB research with respect to rapid sample analysis, cost-effectiveness, minimizing sample exposure to undesired conditions, avoiding extended transporting time to minimize the risk of deteriorating sample quality, and providing adaptability. In the mock test in Takehara Port, the Suitcase Lab performed ideally, including a LAMP assay targeting *A. catenella* in under 2 h after the sample was collected. The time required from sample collection to detect the target phytoplankton species, *A. catenella*, was within 2 h using the Suitcase Lab (Fig. 3). With the current HAB monitoring methodologies, for example, in Chile, the fastest route could still take days from sampling to obtaining microscope analysis data due to the requirement of shipping samples to a laboratory in the complicated geographic area. The ability to detect HAB species at pre-bloom periods will provide advanced warning to the communities who need to initiate mitigation activities such as early harvesting of shellfish and fish or beach closure. With the timely HAB reminder, not only health damages but also economic losses can be reduced for fish farms by stop feeding fish, moving or submerging fish cages, airlifting of deep water into the fish cages, or treating water with flocculants like clay (MacKenzie et al. 2011: Sengco et al. 2001). Thus, effective management of HABs to minimize the effects on coastal communities relies on detecting their development at the earliest possible stage. The Suitcase Lab is a solution to provide field researchers with rapid analysis results using molecular technology. The expensive cost is usually the downfall of using a molecular approach; however, LAMP is relatively cost-effective as one LAMP reaction was estimated at US$ 4.63 for liquid type and US$ 5.83 for lyophilized type (Table 3) and the investment in the Suitcase Lab that enclosed a portable LAMP device was approximately US$ 5500.

Besides cost and time perspectives, the storage unit in the Suitcase Lab was evaluated by testing the storage temperature to maintain samples and reagent appropriately. Our observation showed that the temperature inside the cold box containing the frozen refrigerant was initially − 15 °C and was maintained at below 4 ± 0.9 °C for 17 h when the outside temperature was 25 °C (Fig. S1). This information confirms that materials and reagents required for on-site experiments (e.g., in this study, primers, positive control DNA, nuclease-free water) can be safely stored in the cold box below 4 °C during a 1-day field trip. Maintaining a prolonged cold temperature in the storage box will be one of our challenges to incorporate in the next generation of the Suitcase Lab to be used for more extended field journey as Pomerantz et al. (2018). They were able to transfer materials and reagent from the US to Ecuadorian Chocó rainforest in an engineered food storage container with cold packs and carried out the fieldwork using MinION flowcells.

Although the Suitcase Lab was developed for early on-site HAB detection, its use is not limited to HAB research. The combination of its portability and molecular diagnostic tools can broaden the Suitcase Lab’s potential applications to other research areas especially for epidemiological surveillance in the context of COVID-19, SARS, Chron’s disease, and equine herpesvirus that can benefit from having a portable laboratory (Huang et al. 2020; Poon et al. 2004; Enosawa et al. 2003; Nemoto et al. 2011). The concept of the portable lab has been introduced into many places in the past, such as West Africa and Brazil, where outbreaks of the Ebola and Zika viruses are a matter of serious concern and the use of portable laboratories to analyze the target genome helps in quickly screening viruses during emergencies (Quick et al. 2016: Faria et al. 2017). Its value is highly recognized also in the research fields of ecology, evolution, and conservation for its ability to prevent samples from losing quality associated under intensive field and transportation conditions (Pomerantz et al. 2018: Parker et al. 2017).

Regardless of the Suitcase Lab’s many advantages described above, there are some factors to keep in mind during usage. LAMP assays are limited to qualitative analyses only and can detect a maximum of four target species at a time; therefore, one can only screen for species one is expecting to find with a specific set of primers. These results must be later supported by orthogonal methods that can quantify the target species back at labs (for example, quantitative PCRs or digital droplet PCRs) (Penna and Galluzzi 2013; Eckford-Soper and Daugbjerg 2016b). The enclosed portable microscope (No. 5 and 36, in Fig. 1) can be used but is currently available only for genus identification to collect supportive information on-sites due to its limited magnification (Fig. S2, × 800) and narrow visual field. Upgrading the microscope is one of our objectives to develop the improved version of the Suitcase Lab. Table S1 summarizes the pros and cons of commonly used HAB monitoring strategies. Recently, digital PCR (dPCR) launched as a promising quantitative HAB monitoring tool for its PCR inhibitory tolerance from field samples and its robust and highly reproducible results (Medlin and Orozco 2017: Lee et al. 2017). The combination of tools to be used with the Suitcase Lab is customizable depending on the purposes, monitoring sites, equipment and personnel availability, and budget.

### LAMP specificity and sensitivity

**Fig. 4:**
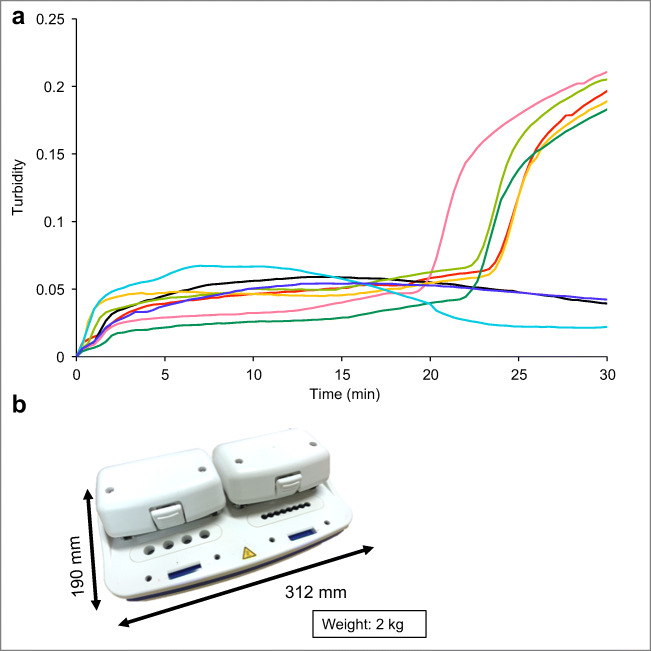
Lamp detection limit for *Alexandrium catenella* cell using *A. catenella* species-specific primers. **a** Turbidities were monitored with a thermostatic color sensor. Red line: with target DNA; black line: negative control; pink line: with *A. catenella* (407.5 cells mL^−1^); yellow line: with *A. catenella* (40.8 cells mL^−1^); light green line: with *A. catenella* (4.08 cells mL^−1^); green line: with *A. catenella* (1.02 cells mL^−1^); light blue line: with *A. catenella* (0.41 cells mL^−1^); blue line: with *A. pacificum* (650 cells mL^−1^). **b** Thermostatic color sensor (KANEKA, Osaka, Japan)

The morphological features of the *Alexandrium* species are difficult to identify even with scanning electron microscopy (SEM) because their differences are subtle, only by the thecal plates (Kim et al. 2017). When John et al. (2014) ran the phylogenetic test on what reported as *A. tamarense* using the information of the rDNA operon, the results actually consisted of five Alexandrium groups, *A. fundyense*, *A. mediterraneum*, *A. tamarense*, *A. pacificum*, and *A. australiense*. Consequently, the taxonomic classification of *Alexandrium* group is kept being changed in the past. This LAMP assay using the gene-specific information can detect *A. catenella* separating from *A. pacificum* and possibly other *Alexandrium* species (Fig. 4a). No signal was obtained for *A. pacificum* indicating that the method was specific for *A. catenella* over *A. pacificum*, the two morphologically very similar HAB species that are almost impossible to distinguish under a regular light microscope (Shin et al. 2017). Alternatively, a future version of the Suitcase Lab with a portable genetic sequencer may identify HAB species with a single test, but this remains to be tested.

The LAMP assay was able to detect *A. catenella* at 1 cell mL^−1^ (Fig. 4a), as can be easily seen in the positive results of target gene detection (Fig. S3). During the *A. catenella* outbreak in Chile occurred in 2009, cell counts at the early bloom stage were estimated as 40 cells mL^−1^, which were gradually increased to 1200, 1500, 1800, 3000, and 6000 cells mL^−1^ in the areas of Cuptana (44° 32′ S), Canalad (44° 33′ S), Angostura (45° 36′ S), Ester (45° 09′ S), and Allan (45° 45′ S), respectively (Mardones et al. 2010). This suggests that the LAMP enclosed in the Suitcase Lab can provide an early warning of a bloom of *A. catenella*.

### Detecting *A. catenella* in natural seawater

**Fig. 5:**
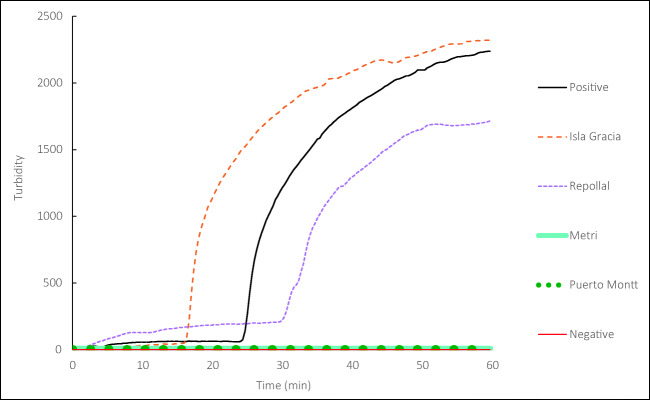
LAMP detection of *Alexandrium catenella* from Chilean coastal waters. Black line: with positive control; red line: with negative control; orange dashed line: with Isla García DNA sample; purple-dashed line: with Repollal DNA sample; light blue line: with Metri DNA sample; green-dotted line: with Puerto Montt DNA sample

The locations RP and IG experienced an *A. catenella* bloom in February 2019. As described in method “Specific detection of Chilean coastal water samples by microscopy and LAMP,” the final volume filtered water was around 1200 L for microscopy and detected *A. catenella* from RP and IG at level 2 (3–10 in 0.1 mL) and 3 (11–42 in 0.1 mL) of 11 levels (174,763–699,050 in 0.1 mL), respectively (see Table S2 and S3). Using the Suitcaase Lab, the LAMP assay also successfully detected *A. catenella* from the same RP and IG samples but from only 250 mL of seawater filtered followed by DNA extraction on site (Fig. 5). The microscopy may have difficulty in detecting HAB species when its concentration is low such as during pre-bloom conditions. However, this LAMP assay is likely to be able to detect *A. catenella* at low concentration as Nagai and Itakura (2012) reported the positive detection result of *A. catenella* from a single cell isolated from culture. Gene detection in environmental samples as compared to in culture samples could be more complicated because they contain a wide variety of microorganisms that can interfere with reactions (Bach et al. 2002); however, the LAMP assay introduced here showed a specific detection of *A. catenella* separating from *A. pacificum*. It confirms that the Suitcase Lab is applicable for *A. catenella* detection from the field samples. One thing to keep in mind is that there is a high risk of cross-contamination on the working space and equipment in the fields by opening the LAMP-product-containing tubes. The result of the assay can be checked without opening it, but if opening is necessary, care must be taken to open the sample containing tubes away from the main station and frequently clean the area to prevent the contamination in the next processing samples. It is our future homework to validate many sets of primers to be specific as much as possible. For instance, it is necessary to check whether *A. catenella* can be separately detected in the presence of *A. ostenfeldii*, which is frequently present in the Aysén region (Salgado et al. 2015; Pizarro et al. 2018). To distinguish toxic HAB species in rapid detection, the primer set to be accompanied by LAMP must be validated prior to use for both culture and environment samples.

## Conclusion

We introduced the development of the Suitcase Lab, a portable laboratory system for rapid detection of HAB species during coastal monitoring. Its applications, however, are not limited to the HAB research but has a greater potential to expand for many other areas who seek a rapid diagnostic tool under geographically limited resources. To the best of our knowledge, this study demonstrated the first molecular approach implemented in Chile for a specific HAB species detection using the LAMP with a portable laboratory. The study successfully showed the productivity of the Suitcase Lab for monitoring *A. catenella*, a well-known HAB species in the field. With the fairly easy operation of the Suitcase Lab, it can be used not only by the trained scientist but also potentially by aquaculturists and fishermen to assess the presence of HAB species.

## Supplementary Information

ESM 1 (DOCX 16 kb)

ESM 2 (DOCX 16 kb)

ESM 3 (DOCX 15 kb)

ESM 4 (DOCX 14 kb)

ESM 5 (PPTX 52 kb)

ESM 6 (PPTX 437 kb)

ESM 7 (PPTX 901 kb)

## Data Availability

All data generated during this study are included in this published article and its supplementary information files.
