# Supplementary material for: Suitcase Lab: new, portable, and deployable equipment for rapid detection of specific harmful algae in Chilean coastal waters
Source: Environ Sci Pollut Res Int. 2020 Nov 18;28(11):14144–55. doi: 10.1007/s11356-020-11567-5 (PMC7673245; doi:10.1007/s11356-020-11567-5)
Supplement: Supplementary file 1 — (DOCX 16 kb) [file 11356_2020_11567_MOESM1_ESM.docx]

**Table S1. Pros and cons of HAB detection methods**

|  | Pros | Cons |
| --- | --- | --- |
| Microscope | - Enable identification and quantitative HAB - Low cost | - Require specialized and trained personnel - The target HAB species cannot be detected at low concentration. |
| Satellite imaging | - Continuous and wide area monitoring - Rea-time monitoring | - Not be able to identify HAB species |
| Molecular method | - Species specific and sensitive detection | - Difficult to transport - Require expensive equipment - Only detect specific HAB species |
| Suitcase lab | - Transportable - Low cost - Species specific and sensitive detection | - Only detect specific HAB species - Qualification application only |
