# Supplementary material for: Suitcase Lab: new, portable, and deployable equipment for rapid detection of specific harmful algae in Chilean coastal waters
Source: Environ Sci Pollut Res Int. 2020 Nov 18;28(11):14144–55. doi: 10.1007/s11356-020-11567-5 (PMC7673245; doi:10.1007/s11356-020-11567-5)
Supplement: Supplementary file 2 — (DOCX 16 kb) [file 11356_2020_11567_MOESM2_ESM.docx]

**Table S2. Relative abundance scales for *Dinophysis acuta*, and *Alexandrium catenella***

| **Description** | **Scale** | *D. acuta^(1)^* | *A. catenella* |
| --- | --- | --- | --- |
| **Absent** | **0** | 0 | 0 |
| **Rare** | **1** | 1 – 5 | 1 – 2 |
| **Scarce** | **2** | 6 – 15 | 3 – 10 |
| **Regular** | **3** | 16 – 35 | 11 – 42 |
| **Abundant** | **4** | 36 - 75 | 43 - 170 |
| **Very abundant** | **5** | 76 - 155 | 171 - 682 |
| **Extremely abundant** | **6** | 156 - 315 | 683 – 2,730 |
| **Hyper abundant** | **7** | 316 - 635 | 2,731 – 10,922 |
| **Ultra abundant^(2)^** | **8** | 636 -1,275 | 10,923 – 43,690 |
| **Mega abundant^(2)^** | **9** | 1,276 – 2,555 | 43,691 – 174,762 |
| **>Mega abundant^(2)^** | **10** | 2,555 – 5,115 | 174,763 – 699,050 |

It is routinely used during the monitoring in IFOP. The average number of cells is counted in 0.1 mL of sample, under the surface of an 18x18 mm coverslip (triplicate).

1. This scale also applies to *A. ostenfeldii*.
2. The relative abundance levels 8, 9 and 10 are exceptional

For more information, visit: <http://ifop.maps.arcgis.com/apps/webappviewer/index.html?id=24ab8163a3034963bc77e44ca015e721>
