# Supplementary material for: Suitcase Lab: new, portable, and deployable equipment for rapid detection of specific harmful algae in Chilean coastal waters
Source: Environ Sci Pollut Res Int. 2020 Nov 18;28(11):14144–55. doi: 10.1007/s11356-020-11567-5 (PMC7673245; doi:10.1007/s11356-020-11567-5)
Supplement: Supplementary file 3 — (DOCX 15 kb) [file 11356_2020_11567_MOESM3_ESM.docx]

**Table S3. Level of *Alexandrium catenella* in February 2019**

| **Location** | **Date** | **Level of *Alexandrium catenella*** |
| --- | --- | --- |
| Metri | 18-Feb-2019 | 0 |
| Puerto Montt | 5-Feb-2019 | 0 |
| Puerto Montt | 12-Feb-2019 | 0 |
| Puerto Montt | 19-Feb-2019 | 0 |
| Puerto Montt | 25-Feb-2019 | 0 |
| Repollal | 5-Feb-2019 | **2** |
| Repollal | 7-Feb-2019 | **2** |
| Repollal | 20-Feb-2019 | **3** |
| Repollal | 26-Feb-2019 | **3** |
| Isla García | 5-Feb-2019 | **1** |
| Isla García | 8-Feb-2019 | **2** |
| Isla García | 20-Feb-2019 | **2** |
| Isla García | 27-Feb-2019 | **3** |
