# Supplementary material for: Suitcase Lab: new, portable, and deployable equipment for rapid detection of specific harmful algae in Chilean coastal waters
Source: Environ Sci Pollut Res Int. 2020 Nov 18;28(11):14144–55. doi: 10.1007/s11356-020-11567-5 (PMC7673245; doi:10.1007/s11356-020-11567-5)
Supplement: Supplementary file 4 — (DOCX 14 kb) [file 11356_2020_11567_MOESM4_ESM.docx]

**Supplemental figure captions**

**Fig. S1 Cold box temperature**

The dotted line (・・・・) shows the external temperature and the dashed line (----) shows the internal temperature. Inside the box the temperature fell rapidly within 5 min and remained below 4°C for 17 h (gray background).

**Fig. S2 Microscopic analysis using the Suitcase Lab**

The diatom were visible at 800 times magnification. However, the genus could not be distinguished.

**Fig. S3 Lamp detection with naked eyes**

The LAMP results could be recognized by naked eyes with either one of three ways; (a) in natural light with white background (change in color for negative samples from orange to green for positive ones), (b) in natural light with black background (appearing white precipitate of magnesium pyrophosphate in the reaction mixture for positive samples), and (c) using a UV lamp.
