# Supplementary figures and images for: Suitcase Lab: new, portable, and deployable equipment for rapid detection of specific harmful algae in Chilean coastal waters
Source: Environ Sci Pollut Res Int. 2020 Nov 18;28(11):14144–55. doi: 10.1007/s11356-020-11567-5 (PMC7673245; doi:10.1007/s11356-020-11567-5)

## Slide 1
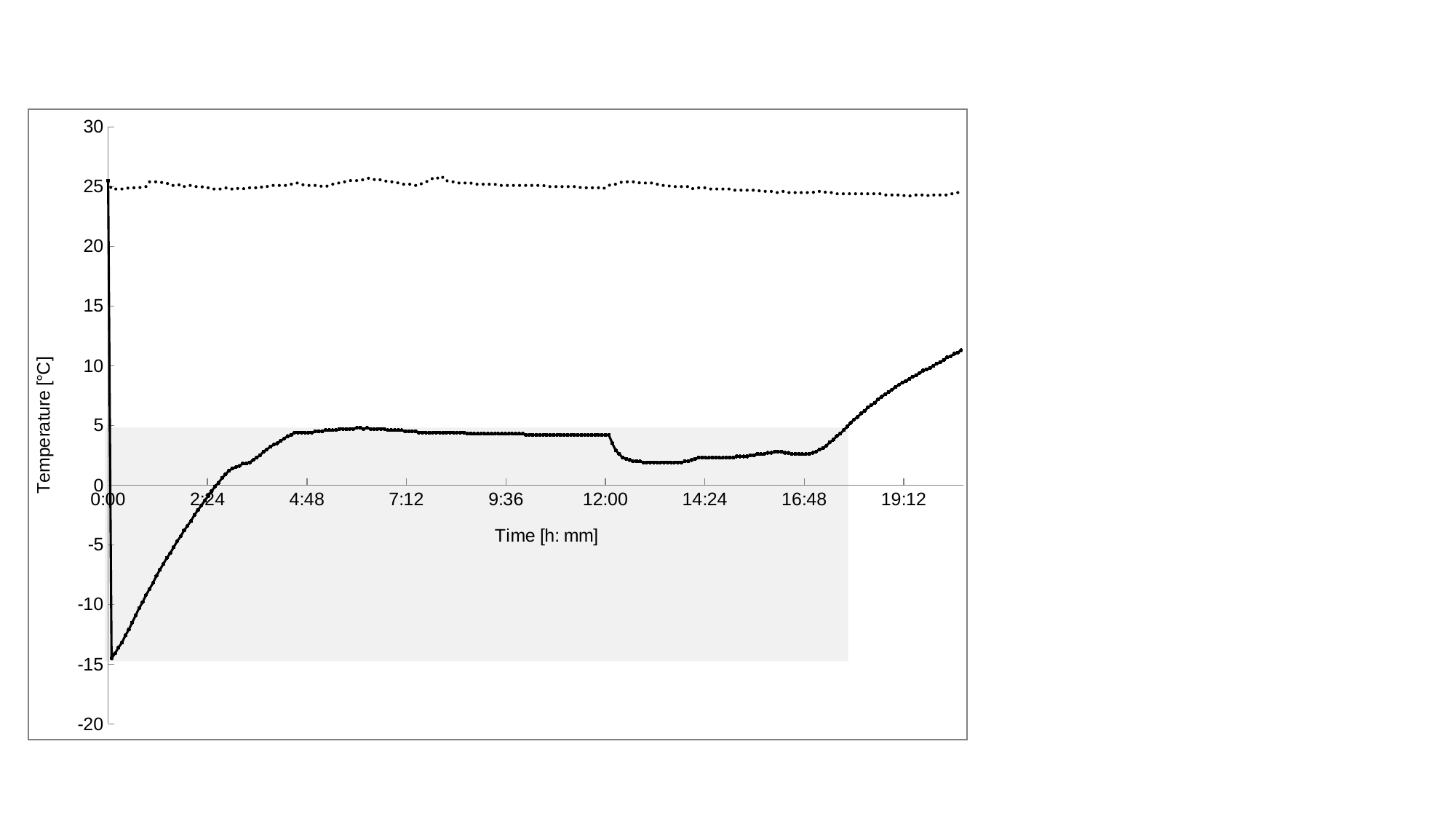

### Chart
| Category | | |
|---|---|---|

Supplement: Supplementary file 5 — (PPTX 52 kb) [file 11356_2020_11567_MOESM5_ESM.pptx]

## Slide 1
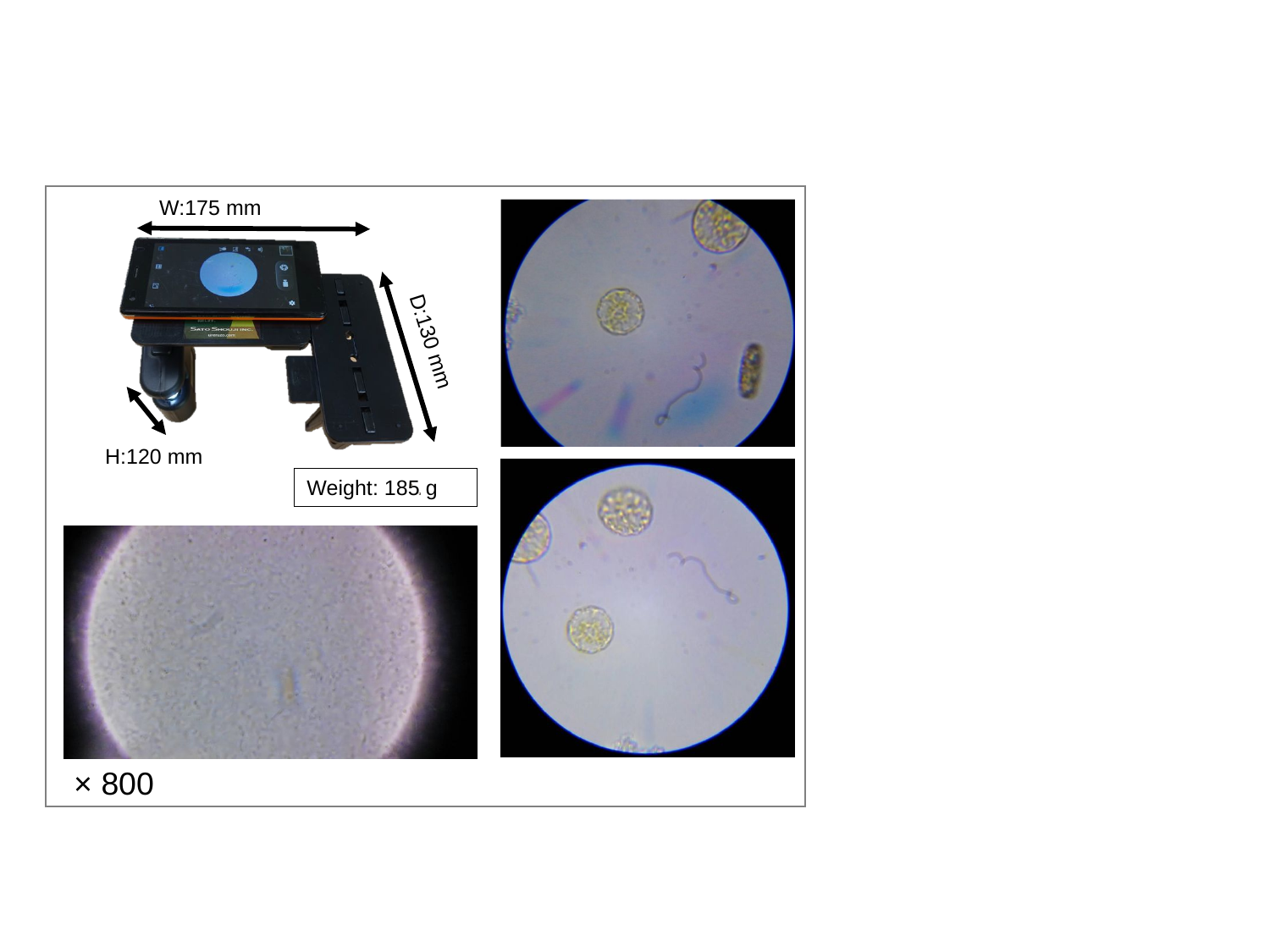

W:175 mm
D:130 mm
H:120 mm
Weight: 185 g
× 800

Supplement: Supplementary file 6 — (PPTX 437 kb) [file 11356_2020_11567_MOESM6_ESM.pptx]
