# Supplementary material for: Suitcase Lab: new, portable, and deployable equipment for rapid detection of specific harmful algae in Chilean coastal waters
Source: Environ Sci Pollut Res Int. 2020 Nov 18;28(11):14144–55. doi: 10.1007/s11356-020-11567-5 (PMC7673245; doi:10.1007/s11356-020-11567-5)
Supplement: Supplementary file 7 — (PPTX 901 kb) [file 11356_2020_11567_MOESM7_ESM.pptx]

## Slide 1
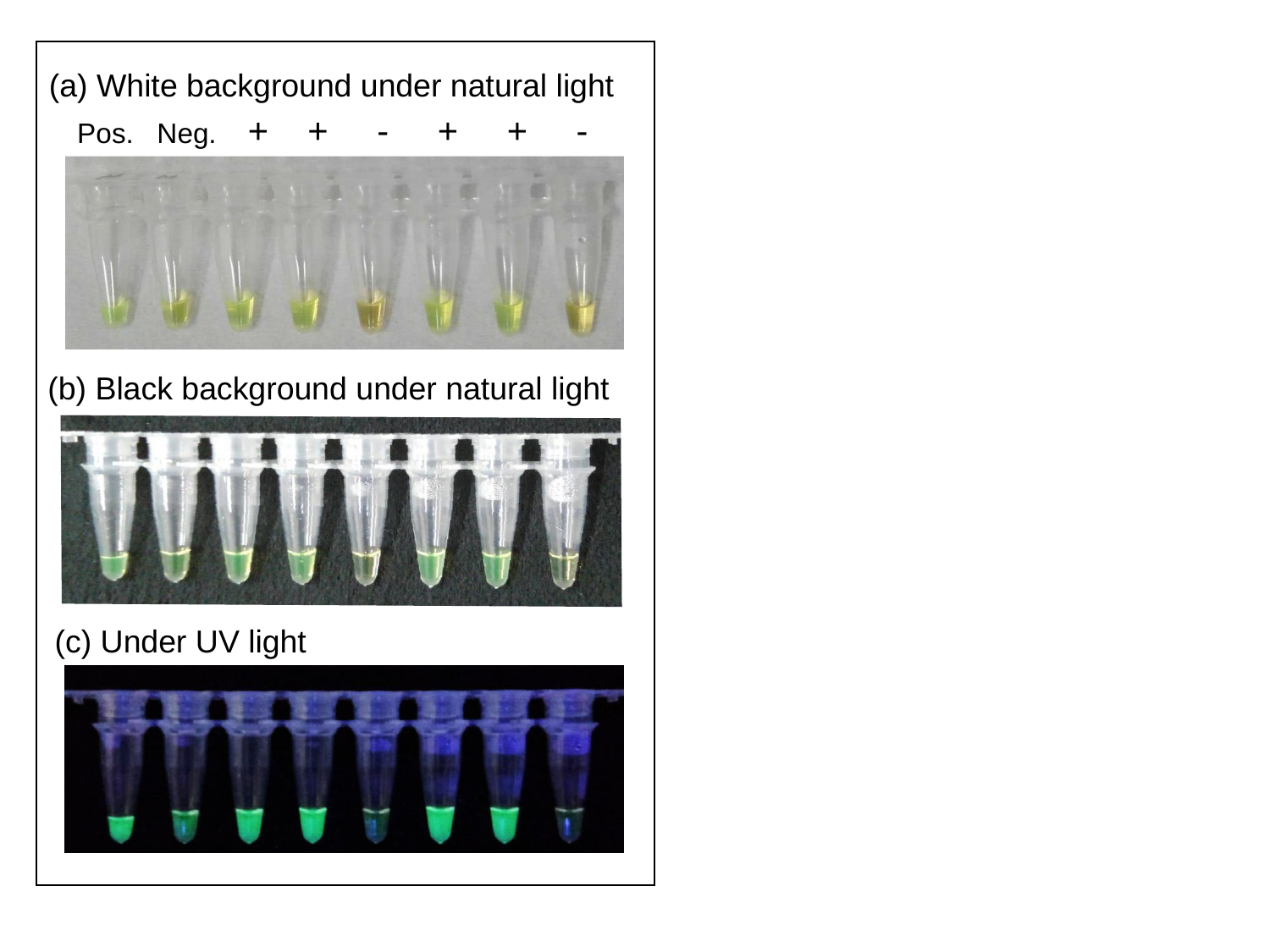

(a) White background under natural light
Pos. Neg. + + - + + -
(b) Black background under natural light
 (c) Under UV light
